# Genito-Urinary and Syphilitic Diseases

**Published:** 1899-11

**Authors:** Byron H. Daggett

**Affiliations:** Buffalo, N. Y.


### Progress in Medical Science.

### GENITO-URINARY AND SYPHILITIC DISEASES.

Conducted by BYRON H. DAGGETT, M. D., Buffalo, N. Y.

### THE CRUSTACEOUS SYPHILIDE.

OHMANN-DUMESNIL (*The Tri-State Jour. and Practitioner*, February, 1899,) says the syphilides present a density of form and appearance and characteristic distribution. For these reasons diagnosis is beset with difficulties for the inexperienced observer.

Quick perception and aptness in observing details are essential in diagnosis of skin eruptions, together with a systematic and thorough examination. Medication should not be depended upon for diagnostic purposes. The term syphilide is applied to all the cutaneous phenomena, due to syphilis; the term syphilodermata being cumbersome we have syphilides, and by analogy tuberculides and lepnides. The term primary syphilis is not applied to the chancre, but to the secondary manifestations, the molecules, papules and pustules and the skin lesions of the tertiary stage.

Among the syphilitic eruptions of the later secondary period of the disease is the crustaceous form, which is not uncommon, and is frequently found in neglected cases, or cases inefficiently treated. The morphology of the crustaceous syphilide indicates that it is the sequence of some anterior stage, such as a pustular lesion or aggregation of lesions. The crustaceous syphilides may be classified as follows : the disseminated, the small and the large aggregated crustaceous forms.

The crust is well formed and thick and its margins are well defined and shapely drawn; it is roundish or ovalish in shape and there is no surrounding ring of pus as in rupia. The margins are closely adherent to the skin, so that forcible removal causes a little bleeding from the skin when the attachment is broken. In color it is a dirty or dark yellow. When the crust is removed another quickly follows. There is a peculiar, penetrating, sickening odor, characteristic of syphilitic lesions, invariably present. This condition is borne of neglect, poverty and squalor, filth and disease. The disseminated crustaceous syphilide vary in size from a dime to a quarter and are very discreet in their distribution. The margins of the crusts adhere closely to the skin. They are prone to occur on the outer aspects of the limbs or the prominences of the trunk. Its distribution shows a notable degree of symmetry. The separate lesions closely resemble each other in size and conformation. The subjective symptoms are nearly the same in the different varieties.

The small, aggregated crustaceous syphilide differs essentially from the other in a number of features. The crusts are quite small, size of a three cent piece to a dime; there is no spreading process. The crusts adhere tenaciously and they form a distinct patch. They are not confluent; each one is formed on a distinct lesion. This form is frequently asymmetrical, for the location may be determined by some local irritating cause. (This is an exception to the rule of symmetrical distribution of syphilitic skin lesions.)

This form may be mistaken for dermatitis, but a history of the case may clear the diagnosis.

The large aggregated crustaceous syphilide is so marked in its appearance and form that it should not be difficult to recognise it. The crusts are as large or larger than the thumb nail and come close together as if developed about a given point. The crusts which form on prominent surfaces thicken from pressure and irritation. This variety occurs in those who are uncleanly, and who are careless in their personal, moral and social habits, addicted to drink and venery. The crustaceous syphilides present no subjective symptoms, except those roused by accidental causes or traumatism. It is a rule almost without exception that no other syphilitic eruption appears upon the integument in the presence of the crustaceous syphilide. If other forms of cutaneous syphilis appear, such as papules or pustules, they form at points distinct from the location of the crustaceous lesion and quickly disappear and abort.

The crustaceous syphilide is never an early manifestation of syphilis, but is an indication of a later process and an evidence that treatment has been inefficient, inactive or not sufficiently prolonged. The characteristics of crustaceous lesions will give data for estimating the age of the disease, as well as some conception of the treatment which has been pursued. Even if papules and pustules develop in the later stages, they present different characteristics than those in recent syphilides. These are called relapsing syphilides; they are more indurated, are less marked in color and less characteristic in form and symmetry than the secondary syphilides.

Treatment should be very active. Give mercauro, beginning with twenty drop doses and gradually increase to thirty, three times daily, if tolerated, and continue for two weeks; then follow up with potassium iodide, beginning with a ʒss (½), and gradually increase to ʒi. Continue this also for two weeks. Alternate these remedies with a week's rest. Locally apply an ointment of :

℞ Hydrarg. oleat, 5 per cent. . . . . . . . . . . . . . ʒii.

Ol. amygdal, dil. . . . . . . . . . . . . . . . . . . . ℥ij.

M. Sig. Three times daily.

A more rapid method is to apply bichlor. hydr. poultices, 1-1000. Wash with sapoderm and powder with nosophen. If an ointment be preferred use :

℞ Ungt. hydrarg. cinerei.
Hydrarg. oleat., 10 per cent. āā.

The prognosis is good under prompt treatment.

METHOD OF USING PROTARGOL IN GONORRHEA.

DR. J. STEPHAN NAGEL, (*The Plexus*, June, 1899,) on the ground of fifty cases successfully treated with protargol, formulates the following general plan of treatment. The protargol injections should be commenced as soon as the discharge appears and continued as long as the microscope shows any of the gonococci present. The treatment should be started with a one-fourth of 1 per cent. solution in distilled water, to be used in a blunt-pointed hard rubber or glass syringe. The patient is instructed to inject one syringeful every four hours day and night and to retain it in the urethral canal by compressing the meatus for two or three minutes. At the end of five or six days the strength of the solution should be increased to one-half of 1 per cent., and during the following five or six days the interval between the injections should be lengthened to six hours, and the solution retained for four or five minutes. The amount of protargol should again be increased to three-fourths of 1 per cent., the interval lengthened to every eight hours, and the period of retention to six or eight minutes. If at the end of the third week a few gonococci are still present, injections of 1½ per cent. should be used twice daily, to be retained in the morning for ten minutes and in the evening for fifteen minutes. In following up this method of treatment an uncomplicated case of anterior urethritis can, in the majority of cases, be entirely and permanently cured in from fifteen to eighteen days.

